# The impact of vascular risk factors on the thickness and volume of the choroid in AMD patients

**DOI:** 10.1038/s41598-021-94676-6

**Published:** 2021-07-23

**Authors:** Elżbieta Krytkowska, Aleksandra Grabowicz, Katarzyna Mozolewska-Piotrowska, Zofia Ulańczyk, Krzysztof Safranow, Anna Machalińska

**Affiliations:** 1 First Department of Ophthalmology, Pomeranian Medical University, Al. Powstancow Wlkp. 72, 70-111 Szczecin, Poland; 2 Department of General Pathology, Pomeranian Medical University, Al. Powstancow Wlkp. 72, 70-111 Szczecin, Poland; 3 Department of Biochemistry and Medical Chemistry, Pomeranian Medical University, Al. Powstancow Wlkp. 72, 70-111 Szczecin, Poland

**Keywords:** Macular degeneration, Retinal diseases

## Abstract

Disturbances in choroidal microcirculation may lead to the onset and progression of age-related macular degeneration (AMD). We aimed to assess changes in the choroidal volume and thickness in the macular region in AMD eyes and to investigate whether coexisting vascular risk factors alter choroidal status. We enrolled 354 AMD patients (175 dry, 179 wet AMD) and 121 healthy controls. All participants underwent a complete ophthalmologic examination and assessment of choroidal thickness and volume. A multivariate analysis adjusted for age, sex, and smoking status revealed that wet AMD was an independent factor associated with higher average thickness of the central ring area (ATC) and average volume of the central ring area (AVC) and lower choroidal vascularity index (CVI) compared to controls (β =  + 0.18, *p* = 0.0007, β =  + 0.18, *p* = 0.0008, respectively) and to dry AMD (β =  + 0.17, *p* = 0.00003 for both ATC and AVC and β =  − 0.30 *p* < 0.0001 for CVI). ATC, AVC and average volume (AV) were lower in AMD patients with hypertension and ischaemic heart disease (IHD). The duration of hypertension was inversely correlated with ATC, AVC and AV (Rs =  − 0.13, *p* < 0.05; Rs =  − 0.12; *p* < 0.05, Rs =  − 0.12; *p* < 0.05, respectively) while IHD duration negatively correlated with AV (Rs =  − 0.15, *p* < 0.05). No such associations were observed in the control group. Our findings show that the choroidal vascular system in eyes with AMD is much more susceptible to damage in the presence than in the absence of systemic vascular disease.

## Introduction

Age-related macular degeneration (AMD) is a progressive disease leading to a severe decrease in visual acuity^1^. Clinically relevant disease is most prevalent in the elderly population, but early signs of AMD can also be found in individuals under 50 years of age^2^. Despite extensive research, the pathomechanism of this disease has not been clarified^3^.

Recently, the role of the choroid in AMD development has been investigated^4,5^. This research was made possible by the introduction of the new technique OCT and enhanced depth imaging (EDI-OCT), which provide better penetration and visualization of the entire choroid up to the uveal-scleral junction^6^. OCT allows noninvasive quantitative assessment of retinal and choroidal structures in vivo. Technological advancements have initiated a new age of research on the role of choroidal microcirculation in healthy, aged, and diseased eyes^7,8^. The choroid is one of the most vascularized tissues in the body. Its main function is supplying oxygen and nutrients to the outer retina, particularly the foveal avascular zone (FAZ), where the choroid is the exclusive blood source for the retina. Recently, the choroid was proven to play a role in the pathophysiology of many vision-threatening ocular disorders, including central serous chorioretinopathy (CSR) and degenerative myopia^9–11^. Choroid thinning is a natural process associated with ageing, but some researchers have shown that in eyes with AMD, this process is much more pronounced^4,12,13^. It is likely that the vascular structure of the choroid undergoes the same pathological changes associated with ageing and the development of atherosclerosis as vessels in other regions^12,13^.

Previous studies have evaluated changes upon CT in both dry and wet AMD; however, the results have been inconsistent^14^. In a study by Jonas et al. that evaluated patients with unilateral AMD, no statistically significant difference between the affected eyes and contralateral unaffected eyes was observed^15^. However, Razavi et al. found CT values to be significantly higher in eyes with neovascular AMD than in unaffected eyes^16^. Notwithstanding, most studies have focused on neovascular or atrophic forms of AMD and have shown that the thinning of the choroid may be a consequence of late-stage disease rather than a cause. A few studies suggested that the thinning of the choroid may originate in the early stages of AMD, although this finding is inconsistent with the results presented in other studies^17,18^. This discrepancy between studies may be partly due to imperfections in the subfoveal CT measurement technique. A single manual CT scan in the subfoveal region can lead to biased results. An analysis of volume based on central 6-mm perifoveal B-scans of the choroid could provide more precise information about choroidal status, as demonstrated in our previous research^19^. An additional valuable parameter recently used in the analysis of choroidal vascularity is the choroidal vascularity index (CVI) obtained by binarization of OCT images^20,21^. The results of the research indicate that CVI is a parameter with lower variability and is influenced by fewer physiological factors compared to the thickness of the choroid, therefore it can be considered a relatively stable parameter for the assessment of changes in choroidal microcirculation^20^. CVI values in patients with age related macular degeneration appeared to be significantly lower in patients with AMD compared to healthy controls^22,23^. In addition, CVI has been shown to decrease with disease duration^24^. Interestingly, either in the study of Koh et al. or Giannaccare et al. subfoveal choroidal thickness did not differ between AMD and healthy eyes, while CVI was significantly lower^22,24^.

Thus, the aim of the study was to assess choroidal volume, thickness and choroid vascular index changes for a detailed analysis of choroidal microcirculation in eyes with dry and exudative AMD. We also investigated whether coexisting vascular risk factors alter the choroidal microvasculature in AMD eyes”.

## Methods

### Subjects and initial management

Patients with a diagnosis of AMD were enrolled in this study. A detailed medical and ophthalmological assessment was provided by the First Department of Ophthalmology of Pomeranian Medical University in Szczecin. We collected data on medical history, current drug use, smoking status and physical activity status from the enrolled subjects, with a particular focus on their history of clinical cardiovascular diseases, including physician-diagnosed heart and vascular diseases. Before the ophthalmic examination, arterial blood pressure was measured (three measurements taken at 5-min intervals gave the mean result) for every participant using a noninvasive blood pressure system with a manual aneroid manometer. Then, the systemic mean arterial pressure (MAP) was calculated: MAP = diastolic BP + 1/3 (systolic BP − diastolic BP) mmHg. We also assessed waist circumference [cm], waist/hip ratio (WHR), and body mass index (BMI) [weight (kg)/height (m)^2^] of all enrolled subjects. To calculate cumulative pack-years, the reported average number of cigarettes smoked per day and the number of years the patient smoked were collected. Finally, with the help of a member of the research team, each participant completed the International Physical Activity Questionnaire (IPAQ), comprising 7 questions regarding all types of physical activity (lasting 10 min or longer) in the previous week. Physical activity scores are presented as MET-min per week and were calculated as described previously^25^. In brief, each activity-specific factor was multiplied by the number of days spent performing the activity and the time in minutes spent performing the activity daily. Weekly activity was measured by adding the score for each of the activities performed.

In accordance with the tenets of the Declaration of Helsinki, a consent form was signed by all patients before trial enrolment.

### Ophthalmologic examination

The patients underwent a complete ophthalmologic examination (biomicroscopic examination of the eye anterior and posterior segments, determination of visual acuity, colour fundus imaging, IOP measurement with a Goldmann applanation tonometer, and axial length and anterior chamber depth calculation). Only eyes with an axial length within the normal range, i.e. 22–25 mm were included in the study^26^. The above mentioned tests were performed to eliminate known factors that interfere with reliable OCT image analysis. If the presence of a neovascular membrane could not be clearly excluded or confirmed, a fluorescence angiography test was performed. Additionally, fundus autofluorescence examination was performed for more accurate drusen visualization to allow correct determination of the AMD stage.

### Optical coherence tomography

Enhanced-depth imaging was performed on both eyes from each subject using the Heidelberg Spectralis SD OCT (870 nm) device (Heidelberg Engineering, Heidelberg, Germany). The A-scan rate was 70,000 scans/s, a light source centred on 870 nm was used, and the axial and transverse tissues resolutions were 3.9 and 6 µm, respectively. To exclude confounding factors, measurements were performed by an experienced technician after 30 min of rest at the same time of the day after pupil dilation with 1% tropicamide solution. In addition, the patients were instructed not to smoke for 6 h or drink any fluids for 1 h before the examination. During scanning, the OCT Spectralis device collected two images using a continuous double laser scan, including an infrared image from a scanning laser ophthalmoscope (SLO) and an OCT scan. The SLO images were used as references for the OCT scans. In addition, a system for actively tracking and correcting eye movements was used. To obtain SD-OCT images of the macular region, a 25° × 25° volume acquisition protocol was used to obtain 49 cross-sectional B-scans. Choroidal segmentation was performed manually after the automated retinal layer segmentation software was disabled. An experienced retina specialist moved the reference lines of the built-in automated segmentation from the retinal boundaries to the choroidal boundaries. This method allowed the use of the automatic retinal thickness and volume map features of the built-in software. The automated software was used to calculate choroidal volume in a similar manner as that used for retinal volume analysis. The details of obtaining OCT images have been described previously^19^.

**Figure 1:**
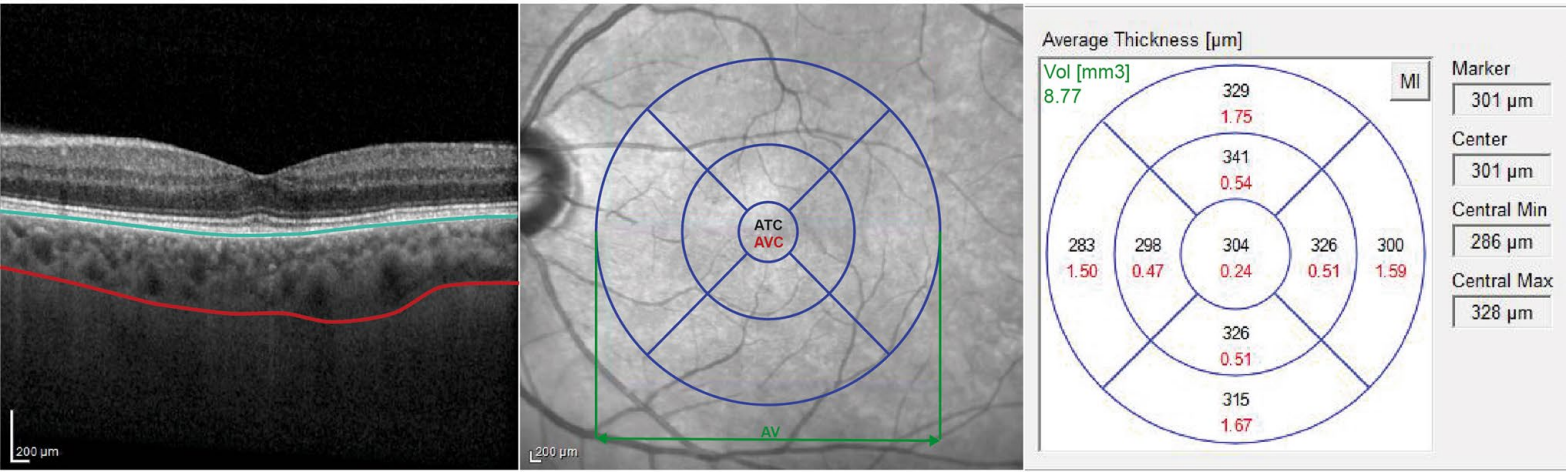
Left: enhanced depth imaging-optical coherence tomography (EDI-OCT) scan of macular region with marked retinal pigment epithelium (blue line) and scleral-uveal junction (red line). Middle: fundus photography with the Early Treatment Diabetic Retinopathy Study (ETDRS) grid applied. Averaged measurements from the central ETDRS region are presented as the averaged thickness center (ATC) and to the average volume center (AVC). Averaged volumetric measurements from the entire area of the ETDRS grid are presented as average volume (AV). Right: the averaged measurements of choroidal thickness (μm) (marked in black) and volume (mm^3^) (marked in red) presented for 9 ETDRS subfields. Values of AV displayed in green.

Choroidal volume measurements were made at all points within the 9 Early Treatment Diabetic Retinopathy Study (ETDRS) subfields, which were automatically provided by Heidelberg Engineering software, were averaged, and are presented as the AV (average volume). Choroidal thickness and volume measurements from the central area according to the ETDRS map were calculated as the ATC (average thickness in central ring area) and AVC (average volume in central ring area) (Fig. 1). For a more precise assessment of choroidal vasculature, we also calculated the choroidal vascularity index (CVI). We used the semi-automated method described previously by Sonoda et al.^27^ with later modifications of Agrawal et al.^28,29^. In brief, EDI OCT scan passing through the subfoveal region was selected and used for analysis. Images with a poorly demarcated choroidal–scleral interface (CSI) were excluded from the analysis. Binarization of the choroidal area in that scan was performed using the ImageJ software (ver. 1.53e; Java 1.8.0_172 (64-bit); https://imagej.nih.gov/ij). The polygon tool was used to select the region of interest (ROI). Next, the selected region was plotted across the entire length of the EDI OCT scan to standardize the area of the ROI among all patients. The upper boundary of the ROI was manually traced along the choroid–RPE junction and the lower boundary along the CSI. Application of auto-threshold was performed after conversion to eight-bit images. Brightness was reduced to allow clear visualization of the choroidal vessels and minimize noise. Niblack’s auto local threshold tool was then applied, to allow demarcation of the luminal or vascular area and the stromal area The image was then converted back to an RGB (red, green, blue) image, and the luminal area was determined using the colour threshold tool. Across all three different thresholding steps (auto-threshold, Niblack’s auto local threshold and colour threshold), we used the default standard settings in the ImageJ plugin that allow us to standardize the thresholding values for all the scans. Choroidal vascularity index was defined as the proportion of luminal area to the total choroidal area and computed for all images. Choroidal thickness, volume and CVI measurements were all performed with one grader. Each measurement was performed three times and averaged. OCT scans in which it was not possible to clearly visualize the choroidal–scleral interface were excluded from the study.

To identify correlations between the stage of the disease and specific systemic and ocular parameters, patients in the study group were assigned to one of three subgroups depending on the status of the disease. Grading of AMD based on evaluation of colour fundus images and OCT using the Ferris classification system was performed by the examining clinician^21^. Group 1 included patients with medium drusen (63–125 μm) but without pigmentary abnormalities, who were considered to have early AMD. Group 2 patients, who had large drusen or pigmentary abnormalities associated with at least medium drusen, were considered to have intermediate AMD. Group 3 included patients with a diagnosis of late-stage AMD including both forms: advanced geographic atrophy and macular neovascularization of any type. Patients with no visible drusen or pigmentary abnormalities and those with small drusen (< 63 μm) were included in the control group because these features are considered signs of the physiological ageing process.

Patients were diagnosed with the dry subform of AMD when there were drusen, pigmentary abnormalities and/or atrophic changes, including geographic atrophy found on colour fundus images and OCT. The wet subform of AMD was diagnosed when the existence of the macular neovascular membrane was confirmed by the presence of intra- and sub-retinal haemorrhages, exudations and/or vascularized PED on colour fundus images, OCT or angiography^3^. All wet AMD patients enrolled in the study were anti-VEGF treatment naïve”.

### Statistical analysis

The Mann–Whitney U test was used to compare quantitative and rank variables between groups. The strength of associations between quantitative and rank variables was assessed by Spearman rank correlation coefficients (Rs). Fisher’s exact test was used to compare qualitative variables between groups. A multivariate general linear regression model (GLM) adjusted for age, gender and smoking status was used to determine independent factors associated with the thickness and volume of the choroid in AMD patients, transformed logarithmically to normalize their distributions. Standardized β coefficients were calculated to show the direction and strength of the associations. A *p* value < 0.05 was considered statistically significant.

### Ethics approval

The study was conducted according to the guidelines of the Declaration of Helsinki, and approved by the Ethics Committee of Pomeranian Medical University in Szczecin, Poland (ethical code is KB-0012/141/13).

### Consent to participate

Informed consent was obtained from all subjects involved in the study.

### Consent for publication

The manuscript has been read and approved by all named authors.

## Results

### Study groups characteristics

We enrolled a total of 354 patients with AMD: 175 with dry and 179 with wet forms of the disease. As a control group, we enrolled 121 healthy subjects. Tables 1 and 2 summarize the clinical characteristics of the AMD patients (divided into dry and wet subgroups) and the controls. Our study groups did not differ in age or the occurrence of well-known atherosclerotic risk factors: hypertension, a history of ischaemic heart disease or myocardial infarction. However, the AMD group had a higher proportion of past smokers and a longer duration of smoking in pack-years than the controls (*p* = 0.0004 and *p* < 0.0001, respectively). We also did not find any correlation between AL and choroidal parameters including AV, ATC, AVC and CVI in wet (Rs =  − 0.21, *p* = 0.65; Rs =  − 0.11, *p* = 0.82; Rs =  − 0.07, *p* = 0.88; Rs =  − 0.03, *p* = 0.93; respectively), dry (Rs =  − 0.39, *p* = 0.13, Rs =  − 0.36, *p* = 0.16; Rs =  − 0.36, *p* = 0.16; Rs =  − 0.02, *p* = 0.95; respectively) and control (Rs =  − 0.55, *p* = 0.1; Rs =  − 0.59, *p* = 0.07; Rs =  − 0.6, *p* = 0.07; Rs =  − 0.16, *p* = 0.68; respectively) study groups.

**Table 1:** Characteristics of the patients in the study groups.

Parameter	AMD group	Control group	*p* value
Number of subjects	354	121	X
Sex (male/female)	135/219	32/89	**0.02**
Age [years] (mean ± SD)	73.4 ± 8.0	73.1 ± 6.0	0.41
Outdoor/indoor working conditions	40.1/59.9%	33.1/67.0%	0.19
Current smokers [%]	13.6%	6.3%	0.0503
Former smokers [%]	51.4%	30.9%	**< 0.001**
Smoking pack-years [mean ± SD]	13.6 ± 18.9	6.0 ± 13.1	**< 0.001**
Period without smoking [years] (mean ± SD)	6.8 ± 10.9	5.3 ± 10.2	0.055
BMI [kg/m^2^] (mean ± SD)	26.9 ± 4.2	26.6 ± 3.7	0.43
WHR [arbitrary units] (mean ± SD)	0.9 ± 0.1	0.9 ± 0.1	0.13
MAP [mmHg] (mean ± SD)	98.3 ± 11.1	98.7 ± 9.7	0.86
Hypertension [%]	64.7%	71.1%	0.27
Duration of hypertension [years] (mean ± SD)	1.2 ± 4.2	9.2 ± 9.9	0.27
History of myocardial infarction [%]	6.2%	6.2%	1
History of ischemic heart disease [%]	16.2%	11.3%	0.33
Duration of ischemic heart disease [years] (mean ± SD)	1.2 ± 4.2	0.8 ± 3.3	0.26
AL [mm]	22.82 ± 1.52	23.13 ± 0.89	0.21

Mann–Whitney U test/Fisher’s exact test. Bolded values indicate significance at *p* < 0.05.

**Table 2:** Characteristics of the patients in the study groups.

Parameter	Dry AMD group	Wet AMD group	*p* value
Number of subjects	175	179	X
Sex (male/female)	52/123	83/96	**0.001**
Age [years] (mean ± SD)	72.7 ± 8.0	74.1 ± 7.9	0.07
Outdoor/indoor working conditions [%]	38.3/61.7%	41.9/58.1%	0.52
Current smokers [%]	10.6%	16.6%	0.14
Former smokers [%]	45%	57.7%	**0.03**
Smoking pack-years (mean ± SD)	10.1 ± 16.1	17.0 ± 20.8	**0.002**
Period without smoking [years] (mean ± SD)	6.3 ± 10.8	7.3 ± 11.0	0.24
BMI [kg/m^2^] (mean ± SD)	26.9 ± 4.3	26.9 ± 4.2	0.99
WHR [arbitrary units] (mean ± SD)	0.88 ± 0.1	0.91 ± 0.1	**0.002**
MAP [mmHg] (mean ± SD)	97.1 ± 10.4	99.5 ± 11.7	0.1
Hypertension [%]	65%	64.4%	1.0
Duration of hypertension [years] (mean ± SD)	8.4 ± 9.6	7.9 ± 9.4	0.64
History of myocardial infarction [%]	7.6%	4.9%	0.36
History of ischemic heart disease [%]	15.1%	17.2%	0.65
Duration of ischemic heart disease [years] (mean ± SD)	1.4 ± 4.8	1.1 ± 3.5	0.59
AL [mm]	23.75 ± 0.97	23.12 ± 0.86	0.38

Mann–Whitney U test/Fisher’s exact test. Bolded values indicate significance at *p* < 0.05.

The proportion of past smokers and the duration of smoking in pack-years were significantly higher in the wet AMD group than in the dry AMD group (*p* = 0.03 and *p* = 0.002, respectively). Furthermore, participants with dry AMD presented lower WHR values than those with wet AMD, which may have been partially related to the difference in gender distribution between study groups.

### Choroid status in AMD and healthy control eyes

The main outcome of the study was to assess the differences in choroidal parameters between the dry and wet AMD subgroups and the control group. Table 3 provides the choroidal thickness and eye volume in the three study groups. We found that both the average thickness and volume in the central ETDRS ring area were significantly higher in wet AMD eyes than in dry AMD eyes (*p* = 0.017, *p* = 0.015, respectively) and control eyes (*p* = 0.022 and *p* = 0.022, respectively). Accordingly, CVI values were significantly lower in wet AMD compared to dry subtype of disease, and values recorded in both AMD subtypes were found to be significantly lower compared to healthy controls”.

**Table 3:** Differences between choroidal parameters in the dry and wet AMD and control groups.

	Control		Dry AMD		Wet AMD		*p* value		
	Median	IQR	Median	IQR	Median	IQR	(Dry vs. control)	(Wet vs. control)	(Dry vs. wet)
AV [mm^3^]	6.96	2.82	6.77	3.55	6.71	2.81	0.641	0.841	0.957
ATC [µm]	273.5	117	269	146	286	161.5	0.907	**0.022**	**0.017**
AVC [mm^3^]	0.21	0.09	0.21	0.11	0.22	0.13	0.937	**0.022**	**0.015**
CVI [%]	0.66	0.03	0.65	0.04	0.64	0.05	**0.01**	**< 0.001**	**< 0.001**

*IQR* interquartile range.

Statistical significance was established at *p* < 0.05 (bolded).

**Figure 2:**
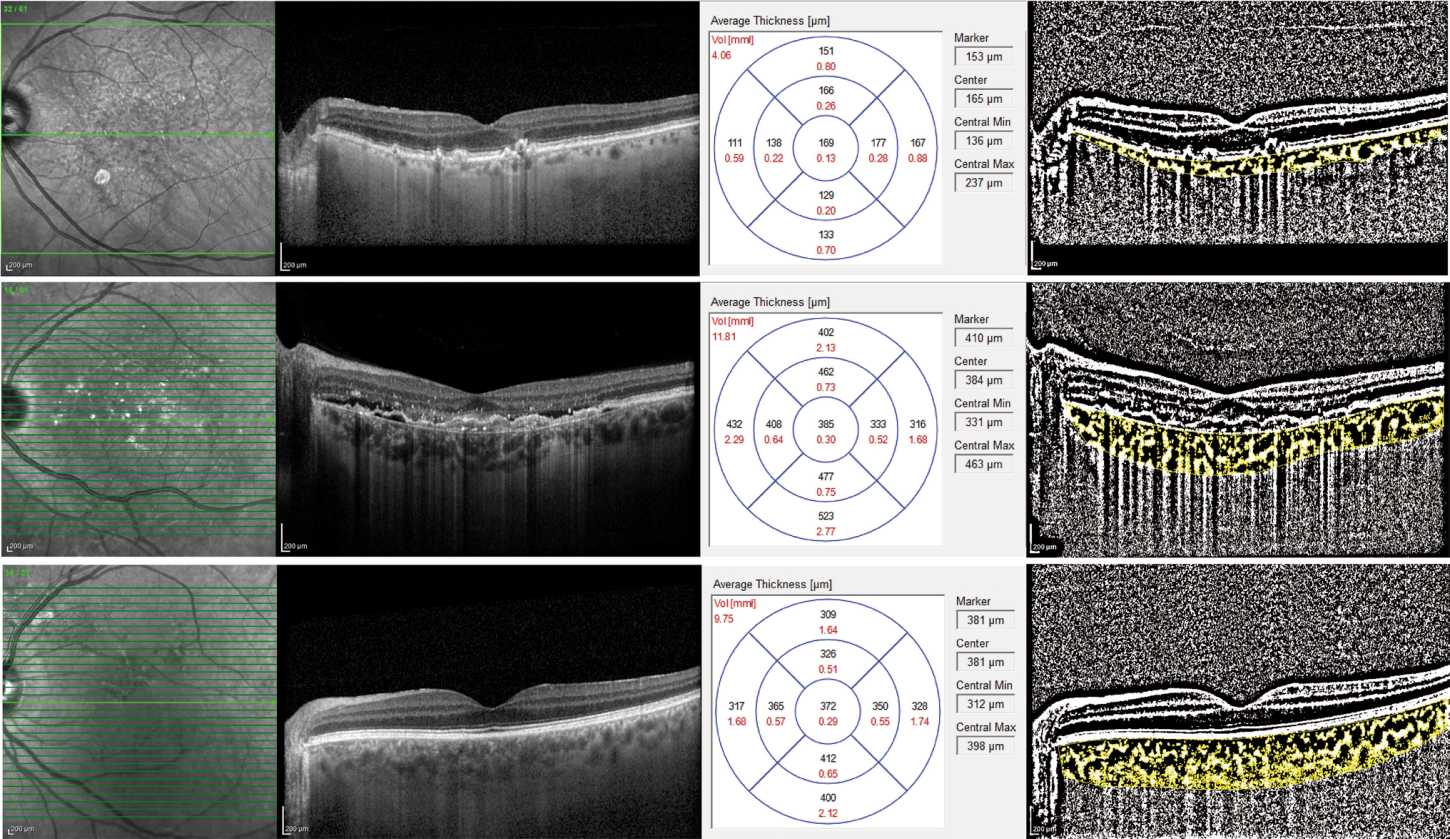
Representative EDI-OCT and CVI scans and averaged measurements of the choroidal thickness and volume in eyes with dry AMD (top line), wet AMD (middle line) and in healthy eyes (bottom line).

Representative choroidal OCT and CVI scans of the patients with exudative AMD and dry AMD are shown in Fig. 2.

A multivariate analysis of patients and controls adjusted for age, sex, and smoking status (pack-years) revealed that wet AMD was an independent factor associated with higher ATC, AVC and lower CVI when compared to controls (β =  + 0.18, *p* = 0.0007, β =  + 0.18, *p* = 0.0008, β =  − 0.42 *p* < 0.0001; respectively) and to dry AMD (β =  + 0.17, *p* = 0.00003 for both ATC and AVC and β =  − 0.30 *p* < 0.0001 for CVI). The covariate most strongly associated with ATC and AVC in these analyses was age (β =  − 0.32 for wet AMD vs controls, β =  − 0.43 for wet vs dry AMD, *p* < 0.000001 for both). The wet and dry groups did not differ in terms of average choroidal volume either between themselves from the control group (*p* = 0.96, *p* = 0.64, and *p* = 0.84, respectively).

In the dry AMD group, choroidal parameters strongly depend on macular morphology and function. Decreases in total choroidal volume, thickness and volume in the central ETDRS area were all associated with lower visual acuity (Rs =  + 0.14, *p* < 0.001; Rs =  + 0.12, *p* = 0.002; and Rs =  + 0.12, *p* = 0.002, respectively). Similarly, CVI correlated with visual acuity in AMD group (Rs =  + 0.19, *p* < 0.001). This may indicate that ongoing atrophy of the choroid in eyes with AMD is associated with a greater degree of functional impairment in the context of AMD. No such correlations were observed in the control group. Accordingly, in dry AMD group we observed negative correlations between the stage of the disease and the AV, AVC and ATC (Rs =  − 0.18, *p* = 0.0004; Rs =  − 0.19, *p* = 0.0002, Rs =  − 0.19, *p* = 0.0003; respectively). Similar association was observed with respect to CVI (R =  − 0.16; *p* = 0.003). It may indicate that choroidal vascular atrophy appears to be more pronounced in the later phases of the disease.

### Correlations between choroidal parameters and the clinical characteristics of the patients

In the AMD group, the age of the participants was strongly negatively correlated with both the choroidal thickness and volume in the central ETDRS area (Rs =  − 0.4, *p* < 0.001, Rs =  − 0.4, *p* < 0.001, respectively) and with the AV (Rs =  − 0.49, *p* < 0.001). A similar relationship was observed in the control group: the age of participants was strongly negatively correlated with the ATC (Rs =  − 0.22, *p* < 0.001), the AVC (Rs =  − 0.22, *p* < 0.001) and the AV (Rs =  − 0.45, *p* < 0.001). In the AMD group, age negatively correlated with CVI (R =  − 0.11, *p* = 0.01), while in the control group, no statistically significant correlation was observed (R =  − 0.006, *p* = 0.93). The above observations confirm the fact that choroidal thinning occurs with age and that the process is more severe in patients with AMD.

Since there is a causative association between vascular diseases and dyslipidemia, we wanted to see if the concentration of blood lipids in specific fractions affects choroid morphology. In the AMD group, the total cholesterol level was positively correlated with the average thickness and volume in the central area and with the total choroidal volume (Rs = 0.14, *p* = 0.02; Rs = 0.14, *p* = 0.02; Rs =  + 0.16, *p* = 0.007; respectively). A similar relationship was shown between all aforementioned choroidal parameters and HDL concentrations (Rs = 0.15, *p* = 0.01; Rs = 0.15, *p* = 0.01; Rs =  + 0.15, *p* = 0.01; respectively). The results suggest that patients with hypercholesterolemia and/or a high HDL concentration have thicker choroids. No such correlations were found in the control group. There was no correlation between choroidal parameters and blood triglyceride concentration in any of the studied groups. Interestingly, choroidal parameters did not statistically differ between statin users and non treated patients.

Because smoking is one of the proven risk factors for the development of vascular diseases, we also evaluated the effect of smoking on parameters of choroid. We considered the duration of smoking, the time since smoking cessation for former smokers, the impact of active smoking and the number of smoked cigarettes expressed in pack-years. Surprisingly, we observed no effect of smoking on measured choroidal parameters in either patients with AMD or in healthy controls.

Since there is evidence that habitual physical activity significantly decreases the risk of vascular diseases, we investigated the association between choroidal parameters and regular physical activity. In the AMD group, physical activity (calculated in MET) appeared to be positively correlated with choroidal thickness and volume in the central ETDRS subfield (Rs =  + 0.13, *p* = 0.04 and Rs =  + 0.12, *p* = 0.04, respectively) and with the average volume (Rs =  + 0.15, *p* = 0.009). There were no such relationships in the control group. We also checked whether obesity affects choroidal parameters and found a negative correlation between CVI and WHR in the AMD group (Rs =  − 0.13, *p* = 0.008).

### Relationship between choroid parameters and systemic vascular diseases

Since available data suggest that choroidal parameters are dependent on underlying systemic vascular disease, we investigated the association between choroidal parameters and several systemic risk factors and conditions. We noted that AMD patients with concurrent vascular diseases had significant reductions in choroidal thickness and volume (Table 4). In the AMD group, patients with concomitant hypertension had a significantly smaller average choroidal thickness and volume in the central ring area and a significantly smaller total choroidal volume than those without hypertension (*p* = 0.001, *p* = 0.002, and *p* = 0.003, respectively). Importantly, the duration of hypertension inversely correlated with both the ATC and AVC (Rs =  − 0.13, *p* = 003; Rs =  − 0.12; *p* = 0.04, respectively) and with the AV (Rs =  − 0.12; *p* = 0.04) which indicates that AMD patients with longer HA durations had thinner choroids. No such associations were observed in the control group. Subsequently, we assessed the effect of concurrent IHD on choroidal parameters in AMD patients and found that those with IHD presented a significantly smaller central average choroidal thickness, volume and total choroidal volume than patients without IHD (*p* = 0.003, *p* = 0.01, and *p* = 0.0006, respectively). Accordingly, in the control group, patients with ischaemic heart disease had significantly lower average choroidal volume parameters than patients without IHD (*p* = 0.05 for AVC and *p* = 0.01 for AV). Importantly, negative correlations between IHD duration and the AV were identified (Rs =  − 0.15, *p* = 0.01), corroborating the observed relationship. Analogous associations regarding a history of myocardial infarction were observed. In the AMD group, patients with MI had significantly lower AVCs and ATCs than patients without MI (*p* = 0.04 and *p* = 0.05, respectively). No such associations were observed in the control group. The results show that vascular disorders affect choroidal flow, as indicated by decreased choroidal thickness and volume, and this phenomenon is much more pronounced in patients with AMD.

**Table 4:** Differences in choroidal parameters between the AMD group and the control group depending on the presence of concomitant vascular diseases and vascular risk factors.

Hypertension (H)	AMD group			Control group		
	H+	H−	*p* value	H+	H−	*p* value
	Median (IQR)	Median (IQR)		Median (IQR)	Median (IQR)	
ATC [µm]	256.5 (119.5)	315 (163)	**0.001**	264 (98.25)	296.7 (109.75)	0.21
AVC [mm^3^]	0.2 (0.1)	0.25 (0.13)	**0.002**	0.21 (0.08)	0.24 (0.09)	0.20
AV [mm^3^]	6.37 (2.85)	6.98 (3.76)	**0.003**	6.56 (2.84)	7.29 (3.65)	0.15

Ischemic heart disease (IHD)	IHD+	IHD−	*p* value	IHD+	IHD−	*p* value
	Median (IQR)	Median (IQR)		Median (IQR)	Median (IQR)	
ATC [µm]	243.25 (89.25)	286 (143)	**0.011**	238.5 (198.5)	280.5 (93.5)	0.057
AVC [mm^3^]	0.19 (0.07)	0.23 (0.11)	**0.009**	0.19 (0.16)	0.22 (0.08)	0.052
AV [mm^3^]	5.97 (1.63)	6.79 (3.33)	**0.003**	5.47 (3.99)	6.98 (2.51)	**0.010**

Myocardial infarction (MI)	MI+	MI−	*p* value	MI+	MI−	*p* value
	Median (IQR)	Median (IQR)		Median (IQR)	Median (IQR)	
ATC [µm]	225 (96.5)	281.75 (141)	**0.045**	248 (79.5)	278 (101.5)	0.49
AVC [mm^3^]	0.18 (0.08)	0.22 (0.11)	**0.042**	0.19 (0.07)	0.22 (0.08)	0.14
AV [mm^3^]	5.81 (1.94)	6.67 (3.16)	0.228	5.86 (2.61)	6.92 (2.91)	0.10

Ischaemic stroke (IS)	IS+	IS−	*p* value	IS+	IS−	*p* value
	Median (IQR)	Median (IQR)		Median (IQR)	Median (IQR)	
ATC [µm]	251.75 (51.5)	280.5 (143.75)	0.435	280.5 (174.5)	270.5 (101.5)	0.48
AVC [mm^3^]	0.2 (0.04)	0.22 (0.11)	0.434	0.22 (0.14)	0.21 (0.08)	0.50
AV [mm^3^]	5.74 (1.01)	7.14 (3.13)	0.275	6.9 (4.56)	6.86 (2.53)	0.41

Limb ischaemia (LI)	LI+	LI−	*p* value	LI+	LI−	*p* value
	Median (IQR)	Median (IQR)		Median (IQR)	Median (IQR)	
ATC [µm]	240.5 (110)	280 (143)	0.587	301.75 (72)	269.5 (121)	0.34
AVC [mm^3^]	0.19 (0.09)	0.22 (0.11)	0.58	0.24 (0.06)	0.21 (0.1)	0.34
AV [mm^3^]	5.82 (2.78)	6.64 (3.09)	0.459	6.63 (1.46)	6.92 (2.74)	0.68

*IQR* interquartile range.

Statistically significant differences with *p* < 0.05 are in bold.

Additionally, we checked whether systemic therapy for vascular diseases influences the choroidal structure. In the AMD group, use of antihypertensive treatment was associated with a lower ATC (hypotensive drug use: median = 250.75 µm, no hypotensive drug use: median = 320.75 µm, *p* < 0.001), AVC (hypotensive drug use: median = 0.2 mm^3^, no hypotensive drug use: median = 0.25 mm^3^, *p* < 0.001) and AV (hypotensive drug use: median = 6.23 mm^3^, no hypotensive drug use: median = 7.05 mm^3^, *p* < 0.001). A similar relationship was identified in the eyes of AMD patients using nonsteroidal anti-inflammatory drugs. Both ATC (NSAID use: median = 244 µm, no NSAID use: median = 287 µm, *p* = 0.004), AVC (NSAID use: median = 0.19 mm^3^, no NSAID use: median = 0.24 mm^3^, *p* = 0.003) and AV (NSAID use: median = 5.99 mm^3^, no NSAID use: median = 6.88 mm^3^, *p* < 0.001) were reduced in patients taking nonsteroidal anti-inflammatory drugs compared to individuals not receiving such therapy. In contrast, no such differences were found in the control group.

## Discussion

Choroidal structure is of particular interest in AMD because abnormalities in choroidal circulation have been hypothesized to contribute to or even precede the development of AMD^5,14^. Some studies suggested that AMD might have a vascular origin, with inadequate choroidal perfusion leading to ischaemia of the retinal pigment epithelium and subsequent production of VEGF, which can result in the formation of a choroidal neovascular membrane^5,30^. This is in line with the results of the current study, which showed that choroidal atrophy increased with age in AMD patients. The reduction in blood flow in the choroidal vessels observed in AMD eyes may be the result of accelerated ageing processes. This leads to narrowing of the choriocapillaris lumen, loss of tissue cellularity, and thinning of the choroid, especially the choriocapillaris layer^13,31^. In the present study, the average values of choroidal thickness and volume in the central ring area of the ETDRS grid in wet AMD eyes were significantly higher than those in both control eyes and dry AMD eyes. Additionally, dry AMD eyes did not exhibit differences in these parameters compared to healthy control eyes. This finding is in agreement with a study by Noori et al., who showed that both the mean CT and CV of the subfoveal area in wet AMD eyes were significantly greater than those in dry AMD and healthy eyes^32,33^. Similarly, Razavi et al. showed thicker choroids in wet AMD eyes than in healthy control eyes in the central ring and in all 9 subfields of the ETDRS grid. This result may be attributed to the presence of oedema, exudation and haemorrhages secondary to the active choroidal neovascular membrane. According to Invernizzi et al., increasing CT in active^34^ neovascularization was secondary to an enlargement of choroidal vessel diameters with or without a corresponding increase in vessel density rather than from tissue thickening secondary to oedema^35^. This might be the result of elevated levels of intraocular VEGF, which leads to choroidal vessel dilation, an increase in blood flow and proinflammatory and proedematic effects^36^. However, in the presence study, CVI was significantly lower in both AMD study groups compared to the control group that confirms the presence of vascular atrophy in AMD. Interestingly, unlike choroidal thickness and volume, the CVI in the wet form of AMD was lower compared to the control group as well as compared to the dry form of the disease. Since the choroidal vascularity index is derived from the ratio of luminal area to total choroid area, a decrease in its value may result from either advanced vascular atrophy or an increase in the volume of all tissue. In the latter case, it may result from the development of the fibrovascular membrane and inflammatory infiltrates in the extravascular tissue of the choroid in response to the increased concentration of proinflammatory and proangiogenic mediators, including VEGF. Other studies assessing choroidal thickness in AMD eyes using OCT imaging have shown mixed results. Chung et al. showed a decrease in choroidal thickness at four points located 1500 µm nasally, temporally, above and below the foveal centre in both dry and exudative subtypes of AMD compared to healthy controls^37^. Similar results were documented by Jonas et al., who showed that CT at a horizontal distance of 1000 µm from the centre of the fovea was not significantly associated with any AMD subtype^15^. It is worth noting that in both studies, point choroidal thickness measurements taken at certain distances from the centre of the fovea were analysed separately (not averaged). CT measurements limited to one or even several points provide a very limited indication of choroidal status. Central choroidal thickness is not, in general, a good predictor of the wider distribution of choroidal thickness on this scale. In contrast, we assessed the averaged thickness parameter obtained from the central ring of the ETDRS grid, which allows the evaluation of the area most frequently affected by the disease in the course of AMD. Moreover, we analysed the average choroidal volume, which allows avoidance of a possible error of a single measurement and errors resulting from the physiological difference in the thickness of the choroid in different quadrants, as the lower nasal sector always presents the lowest value due to the neural tube closure process, while the upper sector, especially the upper temporal sector, physiologically presents the greatest thickness^36^.

The current study showed a negative correlation between average choroidal thickness, volume, choroidal vascularity index and stage of the disease. This indicates a progressive loss of tissue thickness as the disease progresses. Accordingly, in the current study, there was a strong correlation between choroidal thickness, volume as well as CVI and visual acuity. Thus, a smaller choroidal thickness was associated with poorer visual function. Interestingly, Kang et al. found a significant correlation positive between choroidal thickness and visual outcomes in the eyes of patients with nAMD treated with an intravitreal injection of ranibizumab^38^. They concluded that thicker choroids have more potential for recovery due to a relatively preserved choriocapillaris. Similarly, Metelitsina et al. showed that visual loss was associated with decreased choroidal circulation^39^. We would like to emphasize that this is the first study using averaged choroidal thickness and volume measurements to describe the state of the choroid in eyes with different AMD subtypes and at subsequent stages of the disease. This approach provides detailed and reliable information regarding the choroidal parameters of the eyes of AMD patients.

In the present study, we confirmed that concomitant hypertension affected the choroidal microcirculation in AMD patients, leading to the loss of thickness and volume. This leads to the conclusion that the choroidal tissue of AMD patients is more prone to injury due to hypertension than those of healthy eyes. Indeed, the thickness and volume of the choroid in AMD patients in our study was negatively correlated with the duration of hypertension. The results of a study by Ellakwa et al. demonstrated that choroidal thickness was decreased in patients with systemic arterial hypertension. The authors attributed this finding to the process of arteriolar sclerosis and vascular contraction caused by high intravascular pressure in the choroid^40^. Thus, we conclude that prolonged elevated systemic blood pressure impairs compensatory mechanisms and leads to decreased choroidal blood flow, which is followed by ischaemia.

The current evidence suggests that there are multiple similarities between pathogenic mechanisms of AMD and cardiovascular disease^41,42^. However, epidemiological studies have not found a consistent association between AMD and CVD^43–46^. In the current study, patients with AMD and coexisting ischaemic heart disease had significantly lower thickness and volume values than those who did not present CAD. Based on these results, we conclude that the presence of CVD more strongly affects the choroidal microcirculation in patients with AMD than in those without macular degeneration. However, the term CVD generally refers to atherosclerotic disease of medium and large vessels, while the diameter of the choroid is more closely related to the microvessels than to large-diameter coronary vessels^47^. Most likely, the results of our study do not show a simple connection between the two diseases but rather reflect the state of systemic processes associated with biological ageing and other mechanisms underlying the development of cardiovascular disease and AMD. We found that the choroidal thickness and volume were all significantly lower in the eyes of AMD patients receiving antihypertensive and antiplatelet drugs as well as anticoagulants than in the eyes of patients who did not receive such pharmacotherapy. This may suggest that improvements in blood rheological parameters (platelet function) and a decrease in the level of systemic blood pressure or refinement of heart function do not reduce the rate of choroid atrophy in patients with AMD. It is possible that changes in these parameters do not affect the real cause of AMD, which has not been fully established.

There have been a few studies on changes in choroidal parameters directly after exercises in healthy participants, but no studies have analysed the influence of habitual physical activity on choroidal parameters in AMD patients^48,49^. We showed a significant positive correlation between choroidal thickness and volume and physical activity in AMD patients, as patients with AMD who reported more intensive physical activity had a thicker choroid than those with a less active lifestyle. Indeed, an active lifestyle was found to be associated with a lower risk of AMD in several previous studies, probably due to increased antioxidant enzyme activity and increased resistance to oxidative stress, which is thought to be one of the key processes in the pathogenesis of AMD^50–54^.

Because lipids represent at least 40% of the volume of drusen, it can be presumed that lipid metabolism is involved in the pathogenic mechanism of age-related macular degeneration^55^. In this study, plasma levels of total cholesterol and HDL were positively correlated with choroidal thickness and volume. Wong et al. linked the occurrence of a thicker choroid in patients with hypercholesterolaemia and the accumulation of lipids in the suprachoroid region and hypertrophy of endothelial cells and vascular smooth muscle, which was previously observed in animal models^56^. Interestingly, the use of statins did not appear to adversely affect choroidal parameters in our study. This may be due to the pleiotropic effects of statins, which at least partially regulate certain disturbances that contribute to the pathogenesis of AMD.

## Conclusions

AMD is a disease with an unexplained pathomechanism; therefore, effective therapeutic and prevention strategies are still lacking. The results of the current study indicate a relationship between vascular risk factors and choroidal thinning, which suggests an involvement of the choroid in the pathogenesis of age-related macular degeneration. Additionally, our findings show that the choroidal vascular system in eyes with AMD is much more susceptible to damage in the context of coexisting systemic vascular disease. This indicates a double-impact mechanism in which choroids that are altered due to genetic predisposition and/or as the result of certain pathological processes become more susceptible than the choroids of non-AMD eyes to progressive damage resulting from ageing and/or blood circulation disorders.

## Data Availability

The data that support the findings of this study are available on request from the corresponding author, A.M.
